# Role and capacity needs of community based surveillance volunteers in the integrated management of skin neglected tropical diseases (skin NTDs): a qualitative study from central Ghana

**DOI:** 10.1186/s12889-023-16015-w

**Published:** 2023-06-06

**Authors:** Lucy Owusu, Ruth Dede Tuwor, Nancy Ackam, Aloysius Loglo, Bernadette Agbavor, Abigail Agbanyo, Olivia Dornu, Philemon Boasiako Antwi, Michael Ntiamoah Oppong, Jonathan Kofi Adjei, Venus Frimpong, Mohammed Kabiru Abass, Jacob Novignon, Kingsley Asiedu, Dennis Odai Laryea, Yaw Ampem Amoako, Richard Odame Phillips

**Affiliations:** 1 Kumasi Centre for Collaborative Research in Tropical Medicine, Kwame Nkrumah University of Science and Technology, Kumasi, Ghana; 2 Agogo Presbyterian Hospital, Agogo, Ghana; 3 Department of Economics, Kwame Nkrumah University of Science and Technology, Kumasi, Ghana; 4 World Health Organisation, Geneva, Switzerland; 5 Disease Surveillance Department, Ghana Health Service, Accra, Ghana; 6 School of Medicine and Dentistry, Kwame Nkrumah University of Science and Technology, Kumasi, Ghana; 7 Komfo Anokye Teaching Hospital, Kumasi, Ghana

**Keywords:** Community based Surveillance volunteers, Neglected Tropical Diseases, Motivation, Incentives, Sustainability, Ghana

## Abstract

**Background:**

Community Based Surveillance Volunteers (CBSVs) have been instrumental in the management of Neglected Tropical Diseases (NTDs) but a concern that their services in scale up programmes may be affected due to high attrition rates has been widely acknowledged. We explored the roles and capacity needs of existing CBSVs to inform for a successful integrated NTD management programme in Ghana and similar contexts.

**Methods:**

We conducted qualitative interviews with 50 CBSVs, 21 Community Nurses, 4 Disease control officers, 7 skin NTD researchers, 2 skin NTD patients and a Director of District Health Services in Central Ghana. Interviews were digitally recorded, transcribed and coded prior to translation and thematic analysis.

**Results:**

The roles of CBSVs in NTD management were shown to have an impact on disease identification, surveillance, health seeking behaviours and status of CBSVs. Lack of motivation, inadequate structures for engagement of CBSVs within the health system and delayed management of reported cases were identified as gaps that hinder effective delivery of CBSV roles. Provision of incentives as recognition for the unpaid services rendered by CBSVs was seen as a major factor to reduce the rate of CBSV attrition in this scale up programme. Other factors included the formulation of policies by government to guide CBSV engagement, regular training of CBSV in NTD management as well as provision of resources and logistics.

**Conclusion:**

Measures including continuous training, institution of rewards and incentivization are important for ensuring the sustainability of CBSVs in the provision of skin NTD services in Ghana.

**Supplementary Information:**

The online version contains supplementary material available at 10.1186/s12889-023-16015-w.

## Background

Neglected Tropical Diseases (NTDs), as classified by the World Health Organization (WHO) are a group of 20 disease conditions that are most prevalent in the tropical and sub-tropical regions. Globally, more than 1 billion people are affected with the highest case burden found in Africa [1, 2]. NTDs disproportionately affect impoverished populations and lead to stigma, mental distress, deformity and disability in affected individuals as well as accounting for a loss of billions annually to developing countries [1, 3]. A number of the NTDs predominantly occur on the skin, sharing common manifestations and approaches to detection. The WHO recommends an integrated approach to these conditions to facilitate a wider focus on case detection and management, to optimise resource utilization especially in resource-limited regions of the world where the impact of these diseases is greatest [4, 5].

In 2020, the WHO published a new roadmap to guide and direct stakeholder effort in tackling NTDs for the next decade, advocating for a shift from the “siloed disease-specific programs” to a “holistic cross-cutting approach’’. In addition, a training manual has been published by the WHO in this regard [4]. This roadmap for NTDs 2021–2030 highlights the need for prompt and effective diagnosis. Complementary to prompt diagnosis is the need for early case detection through the assistance of community volunteers as has been recommended in Buruli ulcer management [6, 7]. Early recognition and prompt interventions are essential for achieving key WHO milestones as late presentation increases cost and duration of treatment, the degree of deformity and disability along with social stigma in affected individuals [8–10].

Several studies have reported the significant role of community volunteers in the detection, management, control and prevention of various conditions in the health sector worldwide [11–14]. Within the context of NTDs, the roles of community volunteers have been instrumental to the successes of health programs through mass drug administrations, active case search, referrals and surveillance in endemic countries [7, 15, 16].

Community volunteers can be defined as lay members of communities who have received some level of training in order to provide curative or preventive care or control within their communities but are in no wise health professionals [17, 18]. The impact of community-based volunteers is highest in low to middle-income countries, especially in rural Africa where they are involved in a much inexpensive and sustainable patient-centered health care. In Ghana, a community volunteer, known as a Community Based Surveillance Volunteer (CBSV) is a part-time non-paid designation under Ghana’s Community-based Health and Planning Services (CHPS) strategy [19, 20]. In a report on the role of CBSVs in the control of Buruli ulcer in a highly endemic district in Ghana, 70% of BU cases were reported early as a result of CBSV engagement [7] indicating their key role in disease management. In addition, their roles in the control and elimination of NTDs via drug distributions has been highly commendable [21–24].

Notwithstanding, the use of CBSVs in scale up health programmes is costly due to high attrition rates. Factors such as lack of remuneration, recognition and support from communities and government, inordinate amount of time spent volunteering among others, have been frequently highlighted as accounting for the attrition [25–30]. To maximize the impact and sustainability of CBSVs in the integrated management of skin NTDs, it is important to examine roles and capacity needs from their own perspective as well as from the perspective of other stakeholders. Understanding this, will lead to an effective implementation of the programme while minimizing costs associated with identifying, recruiting and training of new CBSVs. Thus, the objective of this work was to contribute to literature on the role and capacity needs of existing CBSVs to make recommendations for efficient execution and sustainability in the integrated skin NTDs management and other prospective programmes within similar contexts.

## Methods

### Ethics statement

The study was approved by the Committee on Human Research, Publication and Ethics (CHRPE) of the Kwame Nkrumah University of Science and Technology (KNUST) with approval number CHRPE/AP/335/19. The ethics approval covered all the study sites. All participants provided written informed consent. All study procedures conformed with the principles guiding research in human subjects as set out in the Declaration of Helsinki [31].

### Study sites

The study was conducted in four NTD endemic districts; Ahafo Ano North and Sekyere East (both in Ashanti region) as well as Asunafo South and Asutifi South (Ahafo region) which are study sites of the Kumasi Centre for Collaborative Research (KCCR) Skin NTDs research group who work with a network of CBSVs for the management of leprosy, Buruli ulcer and yaws within these districts. The districts are located within the central part of Ghana but have not had any significant support from non-governmental organisations for skin NTD activities in the recent past.

### Participant recruitment and sampling

The study used a semi-structured interview guide (Additional file 1) adapted from the construct of the Normalisation Process Theory (NPT) [32] to assess and explain how the work of CBSVs can be nested in community healthcare delivery particularly in the management of skin NTDs. The NPT was adopted as it helps identify societal, policy and health systems factors that can drive effective integration of CBS volunteering into the community health care delivery. Interviews were conducted with fifty CBSVs from 34 communities within the selected districts using purposive sampling technique. In addition, purposive sampling was employed to conduct interviews with twenty-one community nurses, four Disease Control Officers, seven skin NTD researchers, two skin NTD patients and one Director of district health services. Purposive sampling enabled the study team to elicit a diversity of opinions. Data saturation guided the number of participants recruited into this study.

### Data collection

We conducted qualitative interviews over a 3-month period in the year 2020 in selected skin NTD endemic districts in Ghana.

Eight Focus Group Discussions (FGDs) were held with CBSVs and health workers to gather multiple perspectives. Focus groups typically comprised of 8–12 male and female participants who were recruited during a skin NTD workshop organized to train health workers and CBSVs in preparation for a case search exercise within the study districts. Participants were approached and the details of study was introduced to them. Persons who provided informed consent were the recruited to partake in the study. In each of the districts at least one separate focus group discussion was conducted for the CBSVs as well as for the health workers who were community health nurses involved in management of individuals affected by skin NTDs.

In-depth interviews were conducted with other participants including patients, disease control officers and researchers with experiences in community health programmes to gain individual experiences and perspectives in relation to topical areas. A key informant interview with a District Health Director was conducted to add additional insight to the information collected. Interview sessions were all conducted in English except with the CBSVs where majority were in the local dialect, Twi. The interviews were audio recorded on password-protected devices to ensure they were accurately transcribed, and a high-quality recording was obtained.

All interview sessions were conducted in a private room to ensure privacy and lasted for 35–75 min. Topic guides included questions on (i) knowledge assessment of CBSV roles (ii) policy and guidelines defining such roles, (iii) impact and outcomes of community-based volunteering and (iv) factors necessary for sustainability and scale up of CBSV roles. Prior to data collection, the research team received a day’s training, after which the tools were piloted. Only minor modifications such as reframing the questions to reflect the different category of participants were deemed necessary after the pilot.

### Data analysis

All interviews were translated (those conducted in Twi), transcribed in English and were independently verified by 4 research scientists (LO, RDT, PBA and MNO). Transcripts were then assigned unique numbers to ensure anonymity and coded using QDA Miner 4 Lite. The first coding process was done by a team member, LO, to inductively generate the first level topics and segments emerging from the data. These were reviewed by LO and RDT for consistency and credibility. After the initial review, related topics and segments were merged into five broad thematic areas adopted from the NPT during data collection. The coded transcripts were further reviewed by another team member (YAA) for accuracy.

## Results

### Demographic characteristics of community based surveillance volunteers

The demographic characteristics of CBSVs who participated are presented in Table 1. Of the 50 CBSVs, there were 32 males and 18 females; 31 were aged above 54 years. Most (48/50) were married but 2 were single. Majority of the CBSVs (40/50) were farmers, 7 were traders, 2 were teachers and 1 was a tailor. Majority of the CBSVs (37/50) had 10 or less years of experience while the rest (13/50) had been volunteering for more than 10 years.

**Table 1:** Demographic profile of Community Based Surveillance Volunteers (CBSVs)

Characteristic	Frequency, n (%)
**Age**	
25–39	4 (8)
40–54	15 (30)
55–69	31 (62)
**Gender**	
Male	32 (64)
Female	18 (36)
**Occupation**	
Farmer	40 (80)
Trader	7 (14)
Teacher	2 (4)
Tailor	1 (2)
**Marital status**	
Single	2 (4)
Married	48 (96)
Divorced	0
Widowed	0
**Religion**	
Christian	37 (74)
Muslim	5 (10)
Traditional	8 (16)
**Years of experience volunteering (years)**	
< 2 years	9 (18)
2–10 years	28 (56)
11–21	13 (26)

### Thematic areas identified

**Fig. 1:**
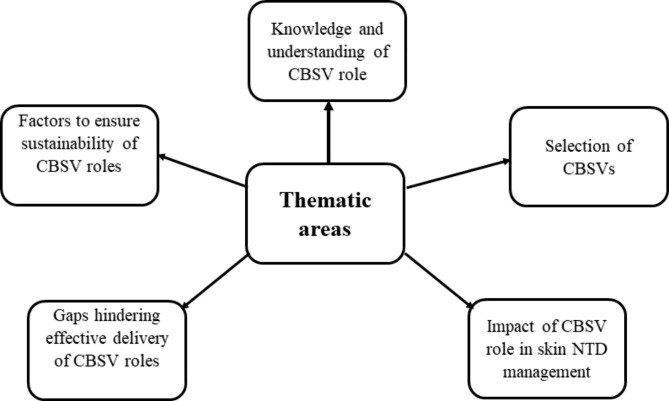
Schematic diagram of thematic areas

From the interviews conducted, related topics were grouped into five broad thematic areas; knowledge and awareness of CBSV role, selection of CBSVs, gaps hindering effective delivery of CBSV roles, impact of CBSV role in NTD management and factors to ensure the sustainability of roles in scale up programmes (Fig. 1). Sub-themes were further examined.

#### Knowledge and understanding of CBSV role

*Perceptions of nurses, health workers, research scientists and patients*.

Participants all agreed CBSV role is key to delivering quality health care. While some participants indicated CBSVs are the first point of call within the healthcare system in communities, others agreed they are crucial in the identification of new cases.

*‘‘Community Based Surveillance Volunteers basically I will say are the eye of health system of the community; they serve as a link between the health authorities and their colleague community members’’.* [*DCO003, Disease Control Officer*]

*‘‘…. the community-based surveillance volunteers are the first point of call for most of our patients, our clients, and participants…you know even the Community Health Worker don’t see the cases unless a volunteer brings them…*’’ [*R001, Researcher*]

*“Initially, I did not know that my child’s condition was buruli ulcer, so what the volunteer did [identification and referral] has really helped us. After the child and I went to the hospital, we saw some other people with the condition which was very bad and they have been in the hospital for a long time, I thanked God that my child’s condition did not get to that stage” [PA001, Father of individual formerly affected by buruli ulcer].*

#### Knowledge and understanding of understanding of CBSV role

*Perception of CBSVs*.

Participants understood their role as being of altruistic nature, desire to educate members of their community and help eradicate diseases.

*‘‘……my understanding of volunteering is that being willing to devote yourself to support the healthcare delivery in the community or willing to support the health care facility to create awareness about health conditions that will improve people’s life in the community…….’’* [*FGDV001, P10 CBSV*]

*‘‘my knowledge about a volunteer is someone who willingly decides to serve the community without any persuasion. And if you willingly decide to do the work you make sure that you are committed to your roles and you do what is expected’’* [*FGDV003, P3 CBSV*].

*‘‘my community members having trust and faith in me that if I need to volunteer in health, I’ll be able to help them. Again, volunteering work is a selfless work that requires hard work and patience without these principles you can’t be a volunteer’.* [*FGDV001, P2 CBSV*]

*‘’…the reason why I have decided to be a volunteer is because the disease we have learnt about now is endemic in the community so it made me devote myself to help so that I can help with the education to reduce the disease occurrence in the community.’’* [*FGDV005, P8 CBSV*]

### Selection of individuals for CBSV role

There were no existing policies regarding CBSV recruitment pathways. CBSVs were selected either by their communities, self-nominations or by health workers in crucial times. Notwithstanding, nominations of persons for CBSV role was premised on factors such as having a good character, willingness to serve and desire to help others.

*“…. One day the health staff in the community came to see our Chiefs if they could nominate someone to serve as a health volunteer in the community. So, the Chiefs called me and another”* [*FGDV001, P1 CBSV*]

*“…. Mostly we leave it to the communities. Unless in critical situations where we fall on our community health nurses. But in the ideal sense, we leave it to the community members because they have their own people. They have their preferred persons”* [*DC003, District Director*].

“*I decided to be a volunteer when I noticed that the way child welfare clinic (CWC) was organized in the community wasn’t satisfactory enough”* [*FGDV002, P5 CBSV*]

*“I took the role of volunteer from my father. He was someone who was committed to the community and voluntarily supported in diverse ways to help with the affairs of health. So, when he became old and was not able to do active work, I decided to take over…….and continue the work of my Dad*” [*FGDV002, P6 CBSV*].

### Impact of CBSV role

CBSV role was found to impact on disease surveillance, health seeking behaviour and case management. The role also had positive impact on the self-esteem of the CBSVs.

“*And if you look at my report, you will realise that, with the Buruli ulcer cases that we have seen, about 90% of them are referred by volunteers. So, you pick the clinic form one by one, and you would realise that nodules and category I cases high. So …early detection is working, instead of later reporting”* [*DCO001 Disease control officer*].

*‘‘When I became a volunteer there were a lot of children with different and several skin rashes on their body, after speaking to the health director to take action, now those rashes have reduced drastically’’* [*FGDV002, P4 CBSV*]

*“..there are quite a number of patient in my community if not for my continuous advice encouragement and support they would have died with their diseases. For example, scabies for instance almost spread across the entire community but with my intervention to report cases and encourage patients to seek care the condition did not spread so widely”* [*FGDV001, P4 CBSV*]

*“I get some fulfilment and dignity for myself, because since I became a volunteer, all health workers in the district know me and even call me by my name. Even when I come to the town or the district capital I see health directors and officers who call me and have some personal discussions with me and it is really an opportunity that gives me self-fulfilment*” [*FGD001, P9 CBSV*]

*“Yes, it is. Had it not been him [community volunteer], we [affected individual and his family] would not have known that it was the buruli ulcer and I would have lost my foot. What he is doing [volunteering] is really important and helpful to us” [PA, 002], former Buruli ulcer patient].*

### Gaps hindering effective delivery of CBSV roles

Many respondents explained that the current structure of the CBSV role within the health system needed to be improved. Gaps highlighted as hindering effective delivery of CBSV roles included lack of motivation, inadequate structures for engagement of CBSV within health system and delayed management of referred cases.

### Lack of motivation

The work of CBSVs requires committing one’s productive hours and resources in serving their communities. Once such a service comes with no motivation, it becomes daunting and does not attract the youth especially in their prime to get involved.

*“…. also because of the remuneration that doesn’t come with it. So, the youth prefer going to work for their money*” [ *DC001, Disease Control Officer*].

*“…Also, one reason why we are all old is that, some of us don’t have a lot of economic activities to do because of our age so we have a bit of time to serve the community but the youth or the young ones are still economically active and will rather prefer to work for financial gain rather than serve the community with no change in their economic lives”* [ *FGDV001, P5 CBSV*].

### Inadequate structures for the engagement of CBSVs within health system

Participants reported that the ad hoc engagement of CBSVs by stakeholders within the health system makes it difficult for their participation in scale up programmes and also accounts for the high attrition rates.

*“As far as I know, I don t think there is a formal structure to sustain CBSVs as part of the Ghana health structure… there are different programs within the Ghana health system, and when they have an activity within the community, then they fall on the services of these community-based surveillance volunteers. So, it becomes a relationship that is more like ‘As and when I need you, I use you’. So, it is not like a permanent sort of relationship that keeps on. Once they have an activity, they fall on the services of the CBSVs and use them for those activities and when the activity ends most at times they are forgotten about.” [R005, Researcher]*

*“…when you train them (volunteers) to do a certain activity and then you forget about them for a period, when you come back to them, even if they have to perform a similar activity or the same activity, they might have forgotten about it because… that is the real difficulty.”* [R005, *Researcher*]

*“…I think one limitation is also not having any reporting form to record and take note of the conditions we find in the community so if such reporting forms to be made available so that as we go for home visits, we can report the conditions we find to enhance our referral system and reporting”* [*FGDV001, P5 CBSV*].

### Delayed management of referred cases

Respondents agreed that delayed responses in decision making by stakeholders on reports submitted by CBSVs sometimes reduces the enthusiasm and zeal of these volunteers in carrying out CBSV roles and eventually leads to withdrawal of services.

*“…However, after referring them to the health facility for about 6 months now nothing has been done to treat the patients. This makes some of them lose trust in the messages and the information and advice which we offer them as volunteers”* [*FGDV002, P6 CBSV*].

*“I am very happy and encouraged when patients I search for are given immediate treatment and becomes discouraged if treatment delays* [*CBSV002, CBSV*].

### Factors for sustainability of CBSV roles

**Fig. 2:**
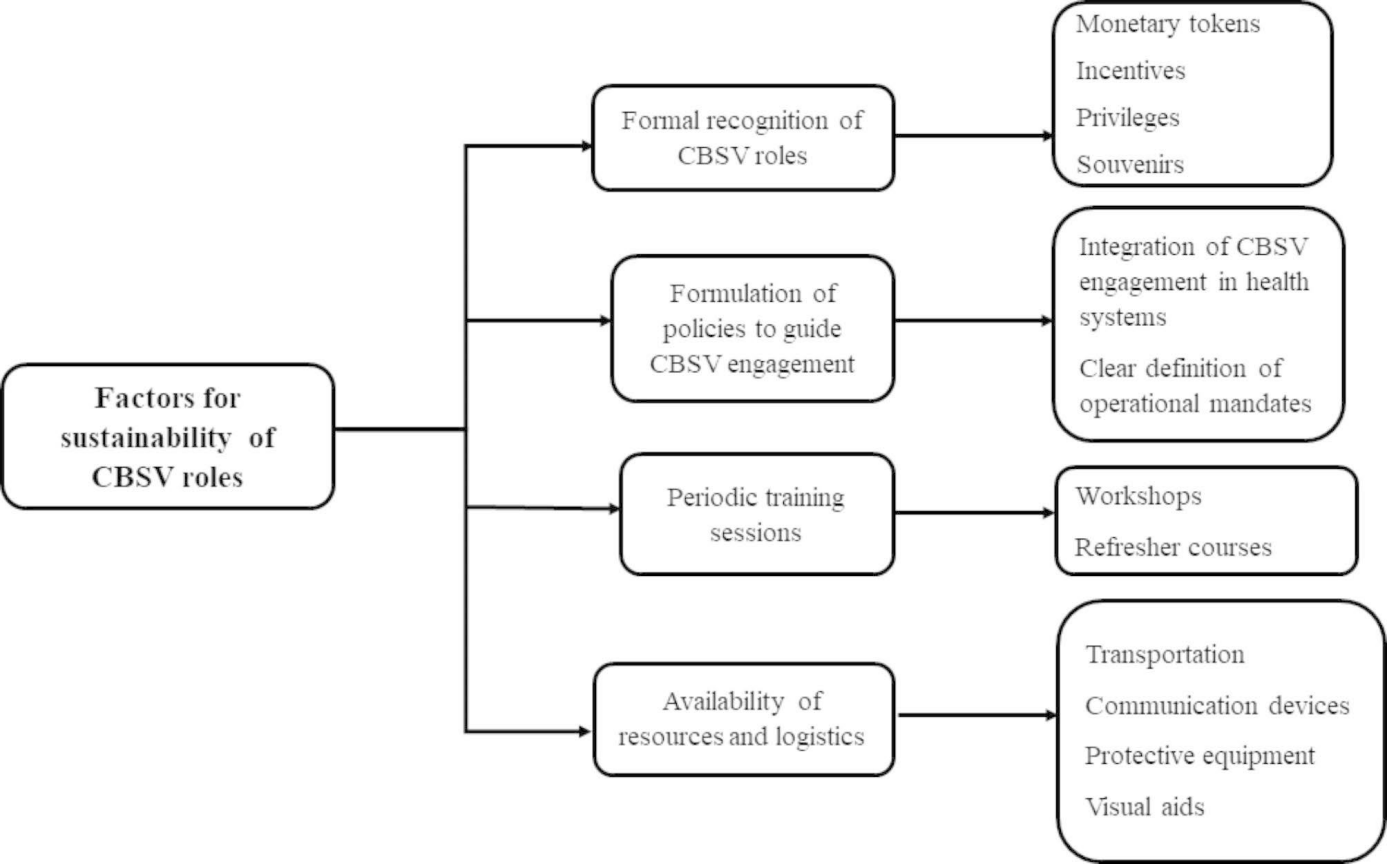
Factors to ensure sustainability of CBSV roles

Respondents enumerated on factors to ensure integration and sustainable contribution of CBSVs in scale up of health programmes. Factors identified were formal recognition of CBSV roles, formulation of policies to guide CBSV engagement, periodic training sessions and availability of resources and logistics (Fig. 2).

### Formal recognition of CBSV roles

Participants agreed the roles of CBSVs demanded committing significant amount of time and energy. Thus, it is important for the efforts of such persons be formally recognized and acknowledged by government and the community through incentives, privileges, stipends among others.

*“Migrating them to a certain level on government pay roll can add some relevance to their roles and I think if there could also be a package per case searched, it will motivate them to bring more cases”* [*FGDHW001 P7, Health worker*].

*“there was a former director who used to organize brief monthly meetings for volunteers, when you close the meeting she gives you a token for transportation which was really motivating because once I know every month I will come to the district for a meeting and I’ll be given either rice or some money I feel excited and I’m always looking forward to attending the meeting and do the work as I’m asked to do”* [*FGDV001, P2 CBSV*].

*“Other incentives could be in the form of in-kind, maybe yearly you celebrate with the volunteers and give them some packages of provisions.”* [*FGDV001, P7 CBSV*]

*“There can also be a policy where every volunteer who goes to the hospital must not join a queue. The volunteer should just show an ID card at the hospital to show that he or she works with the Ghana Health Service as a volunteer and for that matter are not supposed to join a queue, this is motivating enough, and they will feel very appreciated* [*DCO003, Disease Control Officer*].

### Formulation of policies to guide CBSV engagement and define their scope of work

It was agreed that formulation of policies that formally integrates and guides CBSV engagement within the health system is crucial to the sustainability of their roles in health programmes. Participants also indicated the scope of work of CBSVs had not been clearly defined.

*“…… I don’t believe so. It’s not sustainable, especially because these people are not receiving any support, so the temptation is that the free time that he or she has…. he does something, and people can withdraw at any time, because there is no contractual agreement. So, the person decides when to do the house-to-house visit, when to refer, when to do anything related to this volunteering work” [R001, Researcher]*

*“The work does not have limit and some have grown old in the system and since they work with us, they observe and pick up every little thing we do like treatment, so some of them even turn themselves into doctors in the community, so instead of referring the case, they will rather treat the cases in the community and complicate issues. So I believe that with the right policy, we can help them avoid this to make their work more successful*” [*FGDHW001, P2 Health worker*]

*“ I believe that if there is a formal engagement with us where we sign an agreement and some binding rules defining what we do, it can deter some of my colleagues from doing what isn’t expected of them”* [*FGDV002, P6 CBSV*].

### Periodic training sessions

Regular training sessions and refresher programmes for CBSVs was highlighted as a key factor in ensuring the sustainability and maximization of CBSV engagement in integrated health management programmes. While some respondents attested to the impact of regular training has had on case management within their districts, others maintained its absence has reflected on the level of knowledge on CBSVs and their work output.

*“I believe is not sustainable because they need to be constantly involved in the things that they do because the diseases are not going away, and it is not that they have been eradicated. They are still there but the reason is that when you train them to do a certain activity and then you forget about them for a period and then you come back to them even if they have to perform a similar activity or the same activity, they might have forgotten about it because they were only motivated to do it within that specific period of time. So that is the real difficulty”* [*R005, Researcher*]

*“…. I will say no, because I do not think that they have enough knowledge and skills to do what they do, they have not been trained that much to the best of my knowledge because I have been here for 4 years now, and I have never seen any volunteer training in the district or subdistrict specifically on skin conditions. So, there should be some training, if not on monthly basis, quarterly will do. I think that will help.* “[*FGDHW002, P5 Health worker*]

*“early detection is working, instead of later reporting. So, if you compare the two, it means they are doing marvellous work which reflects the training they have receive”* [*DCO001, Disease Control Officer*]

### Availability of resources and logistics

The availability of resources and logistics was identified as key to enable CBSVs continuously perform their roles within the communities. Participants indicated that resources, protective equipment and logistics such as visual and communication aids, means of transportation were needed to support effective functioning of CBSVs. CBSVs indicated the need for provision of protective equipment as most diseases they encounter are communicable and poses a threat to them.

*“So, as I said, the education materials should be available to them and if they can be provided with the implements to make them able to move around within the community, it will help”. [R005, Researcher]*

*“I think he needs a bicycle or a motorbike to enable him move around easily since he is old. Also, he is likely to get tired early if he was to walk from person to person”* [PA002, former Buruli ulcer patient].

*“For me I will stress on the transport logistics because it is a major issue especially with regards to the communities I work. Some places are really far but it’s necessary you visit patient to support them with their medication or check on their treatment but because of the distance it becomes difficult embarking on such journeys.*” [*FGDV001, P2 CBSV*]

*“Sometimes they lack things like Wellington boots and torch lights that they need to work. And so, they complain that they can’t risk their lives to do certain things and then in the long run, they find themselves in other problems. These things sometimes make them opt out of the program”* [*FGDHW002, P9 Health worker*]

## Discussion

Overall, the study identified gaps within the CBSV structure accounting for high attrition and dampening of zeal. Factors necessary to ensure maximum output and sustainability were also outlined. In this study, there were more male than female CBSVs. The higher proportion of males in this study could be attributed to socio-cultural roles of women which may prevent them from participating fully in CBSV roles. Also, the responsibilities of CBSVs requires commuting long distances on foot especially in hard-to-reach communities; given that there are no means of transportation provided for CBSVs, males may be better placed to perform such roles because of their physical conditioning. Our findings are in line with other studies where socio-economic roles were noted to result in the low proportion of women as CBSVs and also account for high attrition rates among women CBSVs [28, 33, 34].

In this study, we noted the impact of CBSV role in four main areas; NTD case identification, management and surveillance, steering positive health seeking behaviours and improving self-esteem of CBSVs. The impact on NTD case identification, management and surveillance was acknowledged by all the stakeholders included in the study. CBSV roles were noted to contribute to early case identification and treatment which is in line with the objectives of WHO roadmap for 2021–2030. In a study by Abass and colleagues, 70% of Buruli ulcer cases were detected early due to the involvement of CBSVs [7]. Through community education activities, CBSVs have played a key role in shaping the health seeking behaviours of community members. Health workers and NTD researchers in our study recounted many accounts of CBSVs contacting and encouraging defaulting patients to continue with treatment. Status and recognition had a positive impact of CBSV role is in keeping with other studies on CBSVs [36–40].

Despite the significant impact CBSV roles on health outcomes, a number of challenges that hindered the efficient delivery of such roles were identified. First, CBSVs acknowledged that their work was voluntary, but desired a formal recognition of their efforts through some form of motivation. In our study, we garnered the role of a CBSV demanded committing significant amount of time to the health needs of their communities. Some CBSVs cited there were occasions where they had to be called upon by community members at odd times (including during the peak hours of their economic activities) which affected their work output. It was intimated that some form of recognition or material benefit for the unpaid services rendered would be appreciated. Also, we observed that majority of CBSVs were within the 55–69 age group. CBSV role requires actively moving through communities which can be an arduous task for persons within this age group and can consequently affect work output. The youth on the other hand, may be better placed to perform such roles. Our study however showed that volunteering was unattractive to the youth, as it was not accompanied by renumeration and other material benefits. We found four (4) kinds of motivation to incentivize the unpaid services of CBSVs; tokens, monetary payments, preferential treatments and souvenirs. Monetary incentives in the form of allowances and stipends dominated as the preferred incentive in this study. A consensus was however not reached as to how much money should be paid as incentive. Again, the issue of standardizing monetary incentive was strongly debated. While some maintained, monetary tokens should be equitably distributed, others argued distribution of such tokens should be based on the operational coverage or number of communities a CBSV was responsible for. Recognition by the community through acts such as ‘gifts of appreciation’, offering assistance with farms or domestic chores, exemption from communal labour and words of affirmation were highly recommended. Singh and colleagues performed a review of the effect of payment and incentives on motivation and focus of Community Health Workers (CHWs) from five low- and middle-income countries (LMICs) [41]. They found community recognition and use of gifts were considered valuable by all CHWs. Preferential treatment given to CBSVs and their family members at health care facilities, job recommendations and admission waivers were noted as instrumental at sustaining interest and thus retention among CBSVs. Lastly, the provision of identification badges, branded T-shirts, caps to CBSVs serve to boost their self-worth by validating them for community recognition and acceptance. Our findings are in keeping with other studies that assessed motivation of CBSVs in Ghana and sub-Saharan Africa [38, 40, 42].

In this study, participants reported an inadequacy of policies to guide CBSV engagement, including recruitment and other operational mandates within the health system. Due to this, the services provided by CBSVs are often regarded to be ad hoc, and volunteers are mainly engaged only during health programmes and disregarded when such programs end, disincentivising volunteers in the process. Furthermore, in the absence of a contractual agreement between CBSVs and the health service, CBSVs intimated they provided services “as and when” they had extra time on their hands to spare. It is therefore important to formally establish policies to integrate CBSV engagement within the health system in order to maximize the effectiveness of their roles.

Some studies [40, 43] have highlighted the lack of clear operational mandates as a challenge to CBSV roles. This study found that some CBSVs were in the habit of treating cases themselves rather than referring to health facilities, thereby complicating management of some disease conditions. Meanwhile, some CBSVs maintained the scope of their work afforded an opportunity to offer some form of curative services (first aid) in instances where health facilities were very distant from communities. CBSVs cited instances where through trainings received, they were able to offer assistance to sick individuals which resulted in their lives being saved. In Ghana, the work of CBSVs spans across disease surveillance, home visits, serving as aids to health officers, maintaining environmental sanitation among others [44]. Within the context of NTDs, they are mostly involved in active case searches, mass drug administrations and community education. It is imperative for the operational mandates of CBSVs to be clearly defined to ensure effectiveness and efficiency of their role in healthcare delivery as has been suggested by Baatiema and colleagues [44].

Non-availability of logistics and resources has been identified to hinder effective delivery of CBSV roles and subsequent withdrawal of services. Logistics and resources were generally grouped into four; transportation, communication devices, personal protective equipment and visual aids. Transportation was mentioned as a major problem responsible for lack of enthusiasm in performing CBSV roles. Although CBSVs were resident in the communities in which they operated, we found that settlements within these communities are far apart. The distance between these settlements without means of transport was intimated to make their work difficult. The provision of “bicycles”, and “transportation allowances” were outlined as sources of motivation for efficient delivery of CBSV role; this is in keeping with other studies that assessed motivation and challenges of CBSVs [38, 45].

Respondents believed that CBSVs and their families are often put at risk since their roles include assessing and referring new cases which may include contagious skin NTDs such as scabies, as personal protective equipment are not supplied. Again, the provision of basic logistics such as raincoats, wellington boots, overalls and flashlights serve to incentivize CBSVs especially in unfavourable weather conditions, thus enhancing their efficiency. The lack of these can cause volunteers to drop out or devote less time to volunteering roles. This is consistent with findings from a multi-country study in Nigeria, Burkina Faso and Uganda [40] where provision of logistics was reported to reduce attrition. Health workers and researchers advocated for the provision of communication devices such as mobile phones and visual aids like posters, flash cards and brochures as these will ease the burden on CBSVs. The incorporation of technology in the management of skin NTDs has been suggested and utilised in some reports [16, 46]. Taking advantage of technology, CBSVs can take pictures of suspected skin conditions from patients in hard-to-reach endemic communities and share with the appropriate experts within the health system for prompt interventions rather than having patients travel over long distances to diagnostic centres (as this can delay treatment). Most skin NTDs can lead to disability, deformity and stigma in the absence of early medical intervention [6, 47]. Also, the use of visual aids by CBSVs can lead to maximization of their efficiency. Researchers interviewed in this study observed that although CBSVs are trained, it is necessary to equip them with visual aids to enhance active case searches within their communities.

In this study, we found adequate training and regular refresher workshops for CBSVs had an impact on their effectiveness and efficiency. In districts where there were regular refresher trainings for CBSVs, there was a positive impact on case referral and management as compared to those where these training sessions were not done. Also, it was garnered that delays in management of referred cases served as disincentive to CBSVs as it resulted in laxity towards performing delegated roles. The issue of case management is however a complex one as it relies heavily on the availability of funds allocated for the management of skin NTDs. When funds are not released on time, there is little that can be done by the health facilities delegated for the management of these diseases.

### Study strengths and limitations

This study has used a qualitative methodology to assess the important role and capacity needs of CBSVs in the delivery of NTD related health services in Ghana. A relatively large number of CBSVs (50) were interviewed. However, there are some limitations. Respondents were only from selected districts in the central part of the country involved in skin NTD services and the findings may not be generalizable to other districts and non-NTD related conditions in Ghana. However, these districts are among the most endemic for skin NTDs and participants have many years of field experience hence the views expressed by participants most likely are reflective of views of CBSVs involved in delivery of services for skin NTDs. Interviews were conducted by research scientists who collaborate with the participating district, so there is a risk that respondents may have provided socially acceptable answers. Further, the responses of participants and nuances may have been lost in translation from the local language, Twi to English; however, this is unlikely given that translation was done by native Twi speakers with several years of experience performing such roles. In all, this study has identified key gaps that when addressed will ensure sustainability of CBSV roles in the integrated management of skin NTDs in Ghana.

## Conclusion

Community Based Surveillance Volunteers (CBSVs) play a vital role that in community healthcare delivery and their integration in the management of skin NTDs is essential particularly in realising the Sustainable Development Goal (SDG) 3.3 on ending the epidemics of neglected tropical diseases and the WHO 2030 skin NTD targets. Our study found that a myriad of approaches including continuous training, institution of rewards system and incentivization are important for ensuring the sustainability of CBSVs in the provision of skin NTD services in Ghana. In addition, the unpaid work provided by CBSVs needs to be assessed as this can inform policy toward achieving the SDG goal 5.4 that calls for the recognition of unpaid work and care through provision of social protection and public services as well as shared responsibilities within the family system.

## Electronic supplementary material

Below is the link to the electronic supplementary material.

Additional file 1: Semi structured interview guides

## Data Availability

The datasets used and/or analysed during the current study available from the corresponding author on reasonable request.

## Abbreviations

NTD: Neglected Tropical Disease
NTDs: Neglected Tropical Diseases
CBSVs: Community Based Surveillance Volunteers
WHO: World Health Organisation
CHWs: Community Health Workers
LMICs: Low- and middle-income countries
KCCR: Kumasi Centre for Collaborative Research
NPT: Normalisation Process Theory
CHRPE: Committee on Human Research, Publication and Ethics
