# Supplementary material for: Role and capacity needs of community based surveillance volunteers in the integrated management of skin neglected tropical diseases (skin NTDs): a qualitative study from central Ghana
Source: BMC Public Health. 2023 Jun 6;23:1086. doi: 10.1186/s12889-023-16015-w (PMC10243008; doi:10.1186/s12889-023-16015-w)
Supplement: Supplementary file 1 — Additional file 1: Semi structured interview guides [file 12889_2023_16015_MOESM1_ESM.docx]

Semi structured interview guide

| **CBSV Demographic data** | |
| --- | --- |
| **Demographic information** | |
| 1. Gender | 1. Male |
|  | 1. Female |
|  | |
| 2. Age | |
|  | |
| 1. Formal education level | 1. Primary |
|  | 1. Secondary |
|  | 1. Tertiary |
|  | 1. Other |
|  | |
| 4. Volunteer experience in months/years | |
|  | |
| 1. Employment status | 1. Student |
|  | 1. Retired |
|  | 1. Working specify (trader, farmer, artisan, mechanic etc) |
|  | 1. Seeking job |
|  | 1. Other |
| Please if other, specify | |
|  |  |
| 1. How were you selected to be a CBSV? | 1. After formal training |
|  | 1. Referral |
|  | 1. Word of mouth |
|  | 1. Self-Nomination |
| Please if specify |  |

| **Domain** | **Questions** |
| --- | --- |
| Knowledge/ understanding of CBSV role | In your opinion, who are CBSVs? |
|  | What do you think is the goal or purpose of the CBSV in the control and management of Skin NTD? E.g., Buruli Ulcer |
|  | How different is the CBSV role in relation to others involved in skin NTD management such as the health care team and clients? |
| Selection of CBSVs | How are CBSVs selected from the community;  Education?  Present occupation?  Self-appointment?  Community nomination?  Gender? |
| Importance of CBSV role | How has CBSV role benefitted the community? |
|  | Has the role benefitted individuals? |
| Challenges faced by CBSVs | What are some of the challenges CBSVs face? |
|  | Do you think there are any drawbacks, threats or risks in CBSV, and if so, what are they?  Do you think CBSVs receive adequate training? |
| Reasons for drop-out | What do you think are some of the reasons why CBSVs drop out of the service? |
| Motivation of CBSVs | What form of motivation can encourage people to become volunteers? |
| Sustainability of role | What can be done to reduce the rate at which CBSVs drop out from providing service? |
